# Social stress increases expression of hemoglobin genes in mouse prefrontal cortex

**DOI:** 10.1186/s12868-014-0130-6

**Published:** 2014-12-04

**Authors:** Adrian M Stankiewicz, Joanna Goscik, Artur H Swiergiel, Alicja Majewska, Marek Wieczorek, Grzegorz R Juszczak, Paweł Lisowski

**Affiliations:** Department of Animal Behavior, Institute of Genetics and Animal Breeding, Jastrzebiec, ul. Postepu 36A, 05-552 Magdalenka, Poland; Faculty of Computer Science, Bialystok University of Technology, Wiejska 45A, 15-351 Bialystok, Poland; Department of Human and Animal Physiology, Institute of Biology, University of Gdansk, 80-308 Gdansk, Poland; Department of Physiological Sciences, Faculty of Veterinary Medicine, Warsaw University of Life Sciences, Warsaw, Poland; Department of Neurobiology, Faculty of Biology and Environmental Protection, University of Lodz, 90-236 Lodz, Pomorska, 141/143 Poland; Department of Molecular Biology, Institute of Genetics and Animal Breeding, Jastrzebiec, ul. Postepu 36A, 05-552 Magdalenka, Poland; iPS Cell-Based Disease Modeling Group, Max-Delbrück-Center for Molecular Medicine (MDC) in the Helmholtz Association, 13092 Berlin, Germany

**Keywords:** Microarray, Gene, Expression, Prefrontal cortex, Social stress

## Abstract

**Background:**

In order to better understand the effects of social stress on the prefrontal cortex, we investigated gene expression in mice subjected to acute and repeated social encounters of different duration using microarrays.

**Results:**

The most important finding was identification of hemoglobin genes (*Hbb-b1*, *Hbb-b2*, *Hba-a1*, *Hba-a2*, *Beta-S*) as potential markers of chronic social stress in mice. Expression of these genes was progressively increased in animals subjected to 8 and 13 days of repeated stress and was correlated with altered expression of *Mgp* (*Mglap*), *Fbln1*, *1500015O10Rik* (*Ecrg4*), *SLC16A10*, and *Mndal*. Chronic stress increased also expression of *Timp1* and *Ppbp* that are involved in reaction to vascular injury. Acute stress did not affect expression of hemoglobin genes but it altered expression of *Fam107a* (*Drr1*) and *Agxt2l1 (Etnppl*) that have been implicated in psychiatric diseases.

**Conclusions:**

The observed up-regulation of genes associated with vascular system and brain injury suggests that stressful social encounters may affect brain function through the stress-induced dysfunction of the vascular system.

**Electronic supplementary material:**

The online version of this article (doi:10.1186/s12868-014-0130-6) contains supplementary material, which is available to authorized users.

## Background

Psychosocial stress affects immune system [1-3], increases the risk of mental health disorders, such as depression [4,5] and anxiety [4], and predisposes to vascular diseases [6,7]. Prefrontal cortex belongs to a neuronal circuitry controlling fear and emotion related behaviors, and is involved in regulation of animal reaction to stressful events [8-10]. The aim of the present study was to investigate transcriptomic changes in prefrontal cortex during stress of social encounter in mice. The main problem in microarray experiments is poor replicability caused by signal noise, fluctuation of gene expression [11-13] and difficulty to select key genes from large amount of transcriptomic data [14,15]. Therefore, we investigated gene expression in 4 groups of mice subjected to acute and chronic stress of different duration in order to find consistent transcriptomic changes that are correlated with duration of stress. In order to check the reversibility of the stress-induced transcriptomic changes, we also determined gene expression after a recovery period following chronic stress. Previously, similar approach but limited to two groups differing in duration of chronic stress has been applied in only two studies investigating brain transcriptome in pigs (frontal cortex and hippocampus) [16] and mice (hippocampal tissue) [17]. Our experiment revealed that expression of hemoglobin genes and *Mgp* was correlated with duration of chronic social stress. Animals that displayed the highest level of hemoglobin genes mRNA had also increased level of genes associated with function of vascular system and injury response. Obtained results suggest that chronic stress may affect brain function through the stress-induced dysfunction of vascular system.

## Methods

### Animals

Ninety-six (8 groups × 12 individuals) Swiss–Webster male mice (weighing 38.3 ± 0.3 g, 12 weeks of age) were used in the microarray experiment and 18 male Swiss–Webster mice (weighing 33 ± 2 g, 9 weeks of age) were used to test the acute effect of stress procedure on blood corticosterone concentration. Additional several cages of group-housed animals (four to five male Swiss Webster mice, 4 months old) were used to stress the experimental mice (see *Social stress procedure* below). Animals were housed in temperature (22 ± 1°C) and humidity-controlled (52 ± 2%) rooms with 12-hour day cycles and provided with ordinary daily care and free access to food and water. Before the start of the experiment mice were housed three to six per cage. All procedures were performed in accordance with the Guiding Principles for the Care and Use of Research Animals and were approved by the Third Local Ethical Committee in Warsaw (permission No. 37/2009).

### Experimental procedure

At the age of 12 weeks the animals that were used in the microarray experiment were moved from their family cages to individual cages and were single housed until the end of the experiment. Immediately after separation mice were assigned to one of the stress group or to the corresponding control group. Next, they were moved from the main colony room to the behavioral laboratory. Mice assigned to stress groups and control groups were kept during the entire period of the experiment in the separate, adjacent rooms. Mice were habituated for 22 days to their new conditions, and next they were subjected to social stress (stress groups) or were left undisturbed (control groups). Mice assigned to the stress group were divided into 4 subgroups (n = 12) described in Table 1. For each stress group, there was a separate control group (n = 12) composed of siblings to enable the comparison between stressed and unstressed brothers. Each stress group and corresponding control group contained at least 10 pairs of siblings derived from different parents. Mice used to test the effect of stress procedure on corticosterone concentration were treated similarly to mice used in the microarray experiment and were singly housed for two weeks before the stress procedure.

**Table 1:** **The design of the microarray experiment**

**Group**	**Habituation**	**Day 1**	**Day 2**	**Day 2 - 4**	**Day 5 - 8**	**Day 9**	**Day 9 - 13**	**Day 14**	**Day 14 - 19**	**Day 20**
		**Stress 1 × 15 min**	**Decap**	**Stress 2 × 10 min**	**Stress 3 × 10 min**	**Decap**	**Stress 3 × 10 min**	**Decap**	**No stress**	**Decap**
Stress 1	V	V	V							
Control 1	V	V	V							
Stress 2	V	V		V	V	V				
Control 2	V	V		V	V	V				
Stress 3	V	V		V	V		V	V		
Control 3	V	V		V	V		V	V		
Stress 4	V	V		V	V		V		V	V
Control 4	V	V		V	V		V		V	V

Decap – decapitation.

V denotes that animals were subjected to specified procedure.

### Food intake measurement

Food consumption was recorded to control the process of habituation and stress procedure. Each mouse received 4 large pellets (about 3 cm long) of standard murine chow. The pellets were weighed and placed on standard stainless steel top grill containing place for food and bottle [18]. Pellets were separated from the bottle by a metal plate to prevent moistening. After 24 hours pellets were weighed again to assess the consumption. Was the pellet about 1.5 cm long or shorter at the time of weighing, it was removed and replaced with a large one. This was done to avoid the possibility that during following 24 h it would become small enough to be pulled into the cage and covered with sawdust. The consumption was recorded from Monday to Friday during habituation period, and from Monday to Sunday during the main part of the experiment.

### Social stress procedure

Social stress was performed by placing an intruder (a stressed animal) into a cage housing four to five male Swiss Webster mice (4 months old). Each session lasted for 10–15 minutes and was performed once or repeated two or three times per day depending on the phase of the microarray experiment (Table 1). Cages with the group-housed mice were rotated after each social encounter. Animals used to test the effect of stress procedure on corticosterone concentration were submitted to a single social encounter lasting for 15 minutes. Animals were observed during the stress procedure to ensure that mice displayed agonistic behaviors such as fights, upright postures, aggressive grooming, and escape [19,20]. Second, behavior was monitored during social encounters to control for the level of aggression and to prevent mice from injuring each other. Because, there was an increase in aggression between mice on the second day of stress procedure, the duration of single encounters was shortened from 15 to 10 minutes. This duration was sufficient to avoid injures. Additionally, number of encounters was increased to prevent potential stress adaptation suggested by a gradual normalization of food intake during the course of experiment. In case of the microarray experiment mice from stress groups and corresponding control groups were decapitated twenty-four hours after the last social encounter. Their brains were removed, while spleens, thymi and adrenal glands were weighed and expressed as percentage of body weight. Changes in the weight of these organs are used as a sensitive measure of stress reactivity in rodents [21-23]. Furthermore, both thymic involution and adrenal gland enlargement are considered to be classic symptoms of stress described originally by Hans Selye [21]. Importantly, these measures of stress reactivity do not interfere with experimental setup. Mice used to test the effect of stress on corticosterone concentration were decapitated 5 minutes after the termination of stress procedure. Immediately before decapitation each animal was individually moved to a separate room designed for dissections.

### Corticosterone assay

Plasma concentration of corticosterone was determined using a high performance liquid chromatography (HPLC) according to modified method described by Ling and Jamali [24]. Blood was collected in 1.5 ml Eppendorf tubes containing EDTA and centrifuged. Corticosterone was extracted from 0.1 ml of plasma samples using ethyl acetate with betamethasone that was added as internal standard. The medium after extraction was centrifuged and the supernatant washed with 0.1 M sodium hydroxide and water. After overnight evaporation of ethyl acetate, the dried samples were dissolved in the HPLC mobile phase, i.e. 35:65 v/v acetonitrile/water. To analyze corticosterone concentration in each sample, we used isocratic HPLC system with UV-DAD detector (Agilent 1100 Series). Detection wavelength was set at 250 nm, RP-C18 analytical column (250 × 3.0 mm, Zorbax, Agilent) and 40°C in column cabinet was kept. Flow rate of mobile phase was maintained at 1.0 ml/min. The extraction efficiency was around 90% and the detection limit of corticosterone was about 1 ng/ml of plasma, with 0.1 ml plasma sample.

### Sample preparation for the microarray experiment

The frontal pole was separated with surgical razor blade in the coronal plane on ice-cold glass dish. Dissected slice (1 mm thick) contained orbital, prelimbic and frontal association cortex located from bregma 3.56 mm to bregma 2.58 mm [25]. This part of brain can be easily recognized because slices consist of the frontal pole and olfactory bulb that are detached from each other. Dissected tissues were inserted into freezing vials, frozen in liquid nitrogen and stored at −80°C until further processing. Total RNA was extracted separately from the individual brain samples using NucleoSpin RNA II kit (Macherey-Nagel, Germany) according to the manufacturer’s protocol. Quantity and quality of RNA samples was estimated using spectrophotometry (ND-1000, Nanodrop, USA) and microcapilary electrophoresis (Bioanalyzer 2100, Agilent, USA). All of the samples chosen for further analysis were of high quality (RIN > 9, 260/280 ~ 2.1).

### Microarray procedures

From each group nine high quality samples that contained the highest amount of isolated RNA were selected. Three independent RNA pools per each experimental group were prepared (24 in total). Each pool consisted of equal amounts of total RNA from 3 mice. Siblings from the stress and control groups were compared on a single microarray. Pairs of animals assigned for each pool were selected randomly. 50 ng of total RNA from each pool was converted to cDNA, then amplified and labeled with cyanine 3 or 5 (Two-Color Low Input Quick Amp Labeling Kits, Agilent, USA). Resulting cRNA was hybridized (Gene Expression Hybridization Kit, Agilent, USA) on Agilent’s Mouse GE 4x44K v2 microarrays. Six slides containing 24 microarrays were used in the experiment. Twelve microarrays were dye-swaps (analyzing the same samples as those in “original” microarray, but labeled inversely) that were included to control the unequal fluorescence of the dyes. Data were extracted using G2565CA Microarray Scanner (Agilent, USA) and Agilent Feature Extraction Software (Agilent, USA) on default settings.

### Real-time quantitative PCR (qPCR)

Microarray data were validated utilizing SYBR Green-based qPCR performed in 96-well plates on the LightCycler® 480 II thermocycler (Roche, Germany). We validated expression level of *Agxt2l1* and *Fam107a* in animals subjected to acute stress, and *Hbb-b1*, *Mgp* and *1500015O10Rik* in animals subjected to 13 days of social stress. Genes selected for validation were representative for clusters with the most consistent pattern of expression across the pools of RNA used for microarray analysis. *Tbp* was used as a reference gene. Total RNA samples from each animal were individually analyzed. PCR validation was extended also to samples omitted in microarray analysis. The number of animals in control groups was reduced to 11 because some samples did not contain enough RNA or did not meet the quality requirements.

Primers (see Table 2 for details) were designed using OligoAnalyzer 3.1. (http://eu.idtdna.com/analyzer/applications/oligoanalyzer/) and Primer-BLAST tools (NCBI, Bethesda, USA) with murine RefSeq database. Primers produced amplicons, which spanned two exons and included all known alternatively spliced mRNA variants. 250 ng of total RNA from each sample was retrotranscribed to cDNA (First Strand cDNA Synthesis Kit, Roche, Germany). qPCR was ran using LightCycler® 480 SYBR Green I Master Kits (Roche, Germany) according to manufacturer’s recommendations. All of the genes were run in triplicate (3 independent runs). All runs contained a negative control (without cDNA) as well as 5-fold dilution series of cDNA to determine PCR efficiency. Melting curve analysis was performed to verify the presence of one gene-specific peak and the absence of primer-dimer peaks. Raw Ct values were calculated on Lightcycler, using 2′nd derivative method. For each sample the relative expression ratio (R) was calculated according to Pfaffl model [26].

**Table 2:** **Sequences, anealing temperatures, efficiencies and amplicon lengths of primers used for qPCR**

**Gene Name**	**Forward or reverse primer**	**Primer sequence**	**Anealing temperature**	**PCR efficiency (%)**	**Amplicon lengths**
*1500015O10Rik*	F	TGGGTCCAGATGGCATAAGTGG	60	82**	105
	R	TTGCTGTGTTCTCGGCTACAG			
*Agxt2l1*	F	GCTCTCCGTTTGCTACTTCAC	60	83*	182
	R	CCCTCTTGACATCTTTGCCCTT			
*Fam107a*	F	CGCTGGTCAGTGTGGTGATT	62	97*	206
	R	AGAGCACCGTCGCAGGAAT			
*Hbb-b1*	F	CTGATTCTGTTGTGTTGACTTG	60	87**	188
	R	AGGTCTCCAAAGCTATCAAAGT			
*Mgp*	F	ACCCTGTGCTACGAATCTCAC	60	95**	140
	R	TTGTTGCGTTCCTGGACTCT			
*Tbp*	F	GCAGTGCCCAGCATCACTATT	60	93*,102**	162
	R	AAGCCCTGAGCATAAGGTGG			

* - PCR efficiency in “acute stress” time point analysis, ** - PCR efficiency in “13 days of stress” time point analysis.

### Data analysis and statistics

#### Stress parameters and real–time quantitative PCR

Weight of body and internal organs, food intake and corticosterone data were subjected to one-way analysis of variance followed by Levene’s test of variance homogeneity. Data that did not meet the requirement of variance homogeneity were subjected to a square root transformation and then were analyzed again following the data normalization guidelines [27]. PCR data were initially subjected to one-way ANOVA followed by Levene’s test of variance homogeneity. Because not all PCR data met the requirement of variance homogeneity, the nonparametric Wilcoxon signed-rank test was applied [28,29]. Relationship between the weight of organs and gene expression was assessed with Pearson Correlation coefficient (normally distributed data) or with Spearman’s rank correlation coefficient (data that were not distributed normally). Normality of data was assessed with Lilliefors test. Statistical analysis was performed with Statistica software, release 7.1. Values are presented as mean ± SEM.

### Analysis of microarray data

#### Statistics

Raw data files were analyzed with the Limma package from Bioconductor [30] using the same criteria for all files. Background correction method “normexp” [31] was used and followed by within-array normalization carried out with the “loess” procedure and between-array normalization conducted with the “Aquantile” method [32]. Genes showing logarithmic fold changes greater than 0.5 (logFC > 0.5) and adjusted p-values less than 0.05 (*p* < 0.05) were considered differentially expressed. Benjamini and Hochberg method controlling for false discovery rate [33] was used for p-value adjustment.

### Clustering analysis

Genes that were found to be significantly up- or down-regulated were included in clustering analysis. For these genes logarithmic fold changes (logFC) for each technical replicate (original array + dye-swaped array) were calculated. LogFC values of different probes for the same gene were averaged using median. These values were used as an input for clustering. Clustering was carried out with the use of the Cluster 3.0 software (Stanford University, USA) and results were visualized in Java TreeView [34]. UPGMA clustering algorithm with absolute centralised correlation as a similarity matrix was used [35]. Correlation coefficient (r) > 0.5 was used to assign genes to different clusters.

## Results

### Stress indices

**Figure 1:**
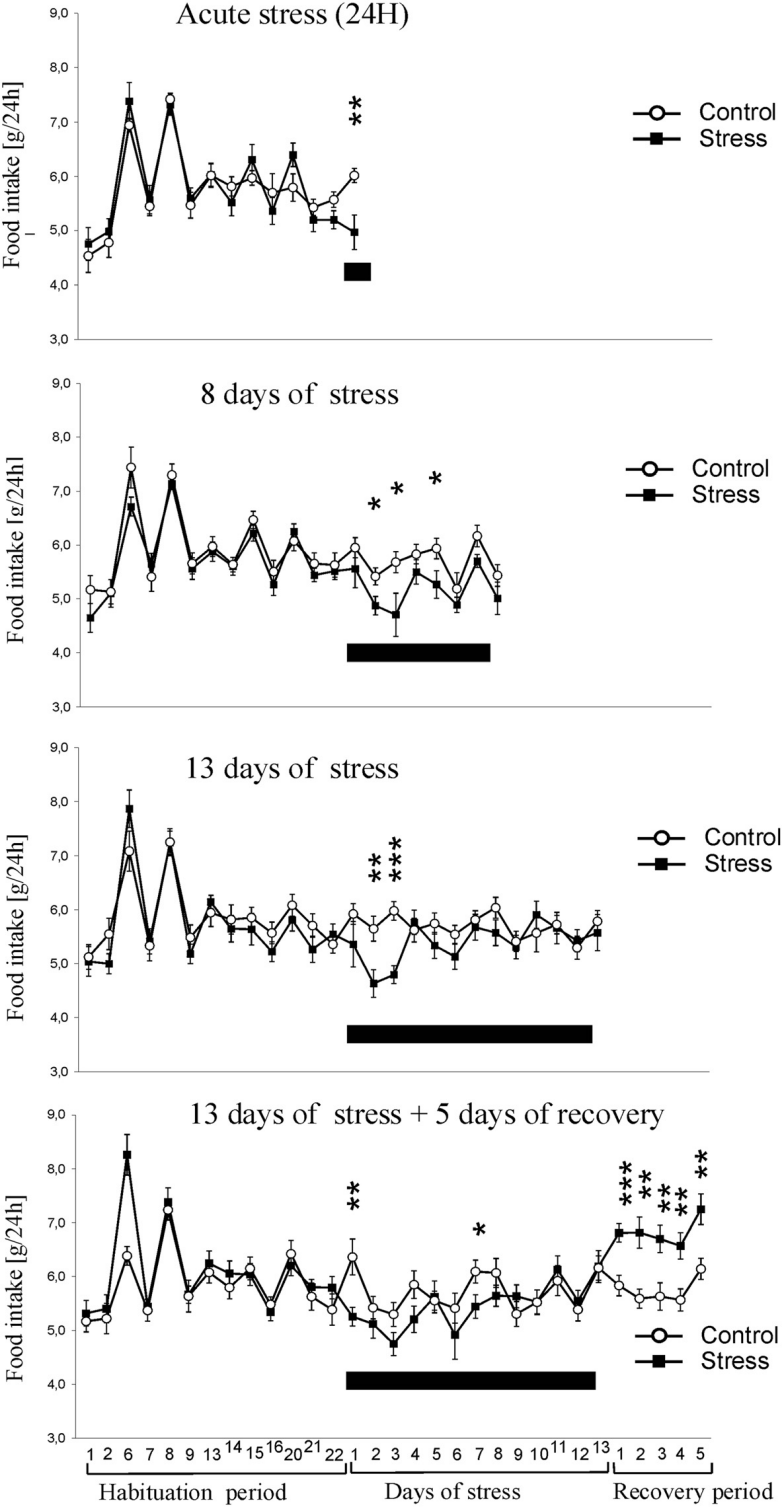
**Effect of stress on food intake.** Black bar depicts duration of social stress. Values are presented as mean ± SEM. N = 12, * - p < 0.05, ** - p < 0.01, *** - p < 0.001; compared with corresponding control group.

**Figure 2:**
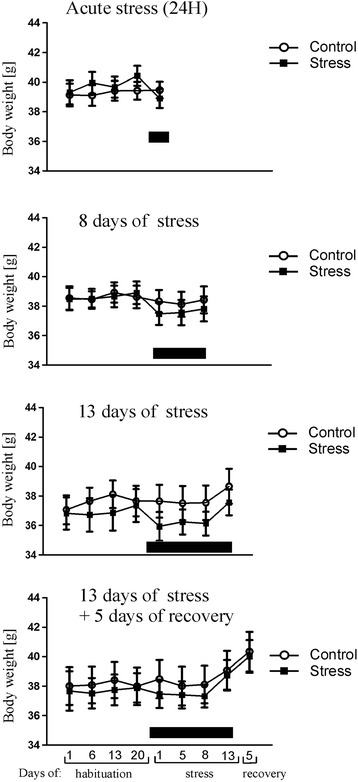
**Effect of stress on total body weight.** Black bar depicts duration of social stress.

**Figure 3:**
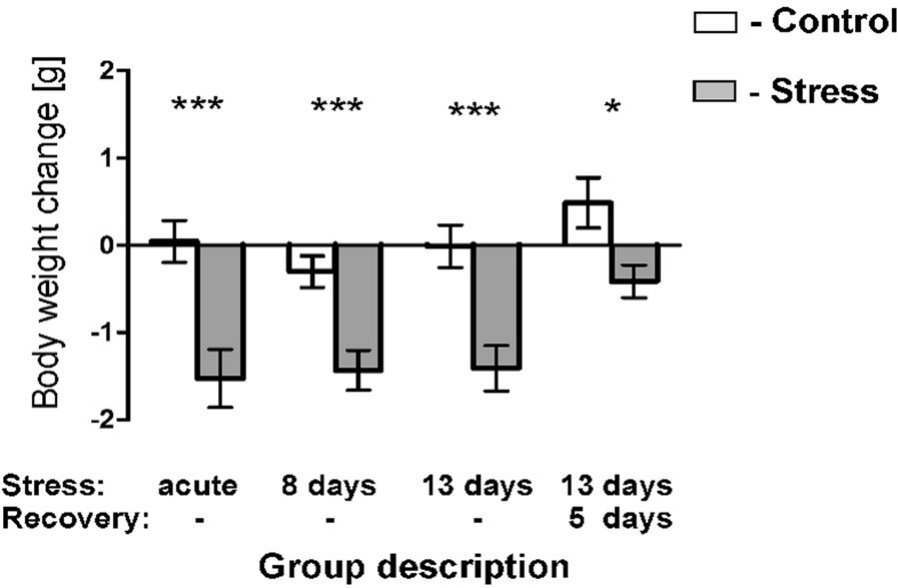
**Body weight gain 24 h after the first social encounter in all four groups used in the microarray experiment.** Values are presented as mean ± SEM. N = 12,* - p < 0.05, ** - p < 0.01, *** - p < 0.001; compared with corresponding control group.

**Figure 4:**
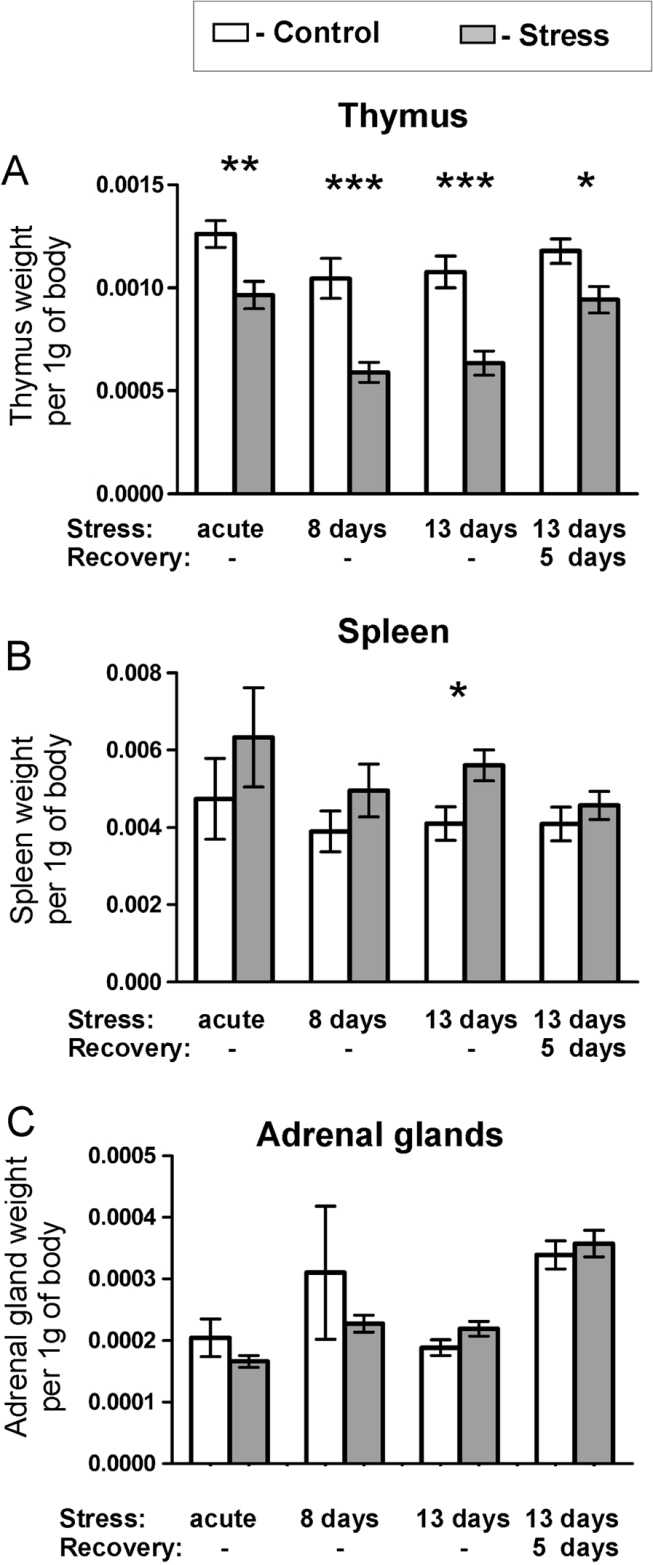
**Effect of stress on the weight of thymus (A), spleen (B) and adrenal glands (C).** Values are presented as mean ± SEM. N = 12. * - p < 0.05, ** - p < 0.01, *** - p < 0.001; compared with corresponding control group.

**Figure 5:**
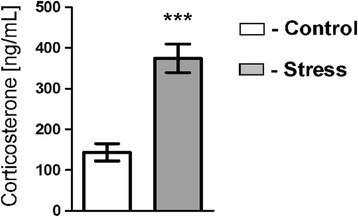
**Effect of a single social encounter lasting for 15 min on blood corticosterone concentration measured 5 min after termination of stress.** Values are presented as mean ± SEM. N = 9; *** - p < 0.001.

Mice displayed cyclic fluctuation in food intake after separation from littermates (Figure 1). These fluctuations were associated with the cycle of work of personnel responsible for maintenance of the mouse colony. Cyclic changes in food intake stabilized during the period of habituation. Social stress significantly decreased food intake in all stressed groups (Figure 1). After several days of repeated social stress, food intake returned to the baseline and then increased during the recovery period (Figure 1). Total weight of mice has not been significantly altered by stress (Figure 2) but there was a significant decrease in body weight gains in all stressed groups 24 h after first social encounter (Figure 3). Thymi were significantly lighter and spleens were significantly heavier in the stressed animals (Figure 4A and B). In case of adrenal glands the results were characterized by lack of stable baseline and large differences in variability between groups (Figure 4C). High variability could result from difficulty to precisely separate small adrenal glands from surrounding adipose tissue. Differences in weight of adrenal glands were insignificant although the p value approached the level of significance in case of animals subjected to 13 days of stress (p = 0.09). In a separate experiment it was found that a single social encounter with a group of mice induced large increase in blood corticosterone concentration 5 minutes after the termination of stress procedure (Figure 5).

### General genome-wide expression

**Figure 6:**
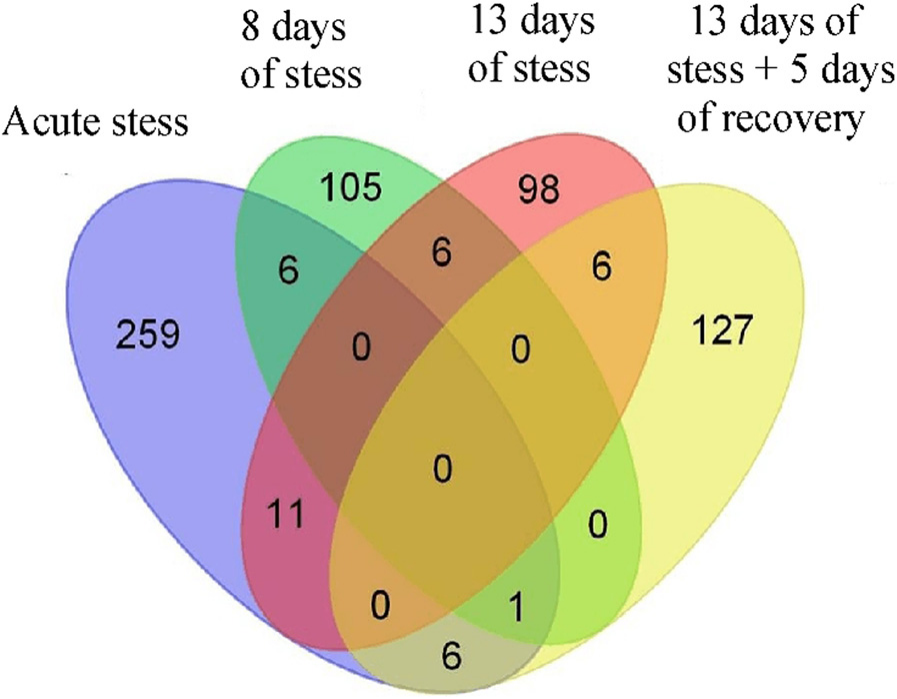
**Venn diagram showing differences and similarities in gene expression between different stress regimes.** Each colored ellipse represents one treatment group. Numbers of genes common between treatment groups are depicted on intersections between ellipses.

**Figure 7:**
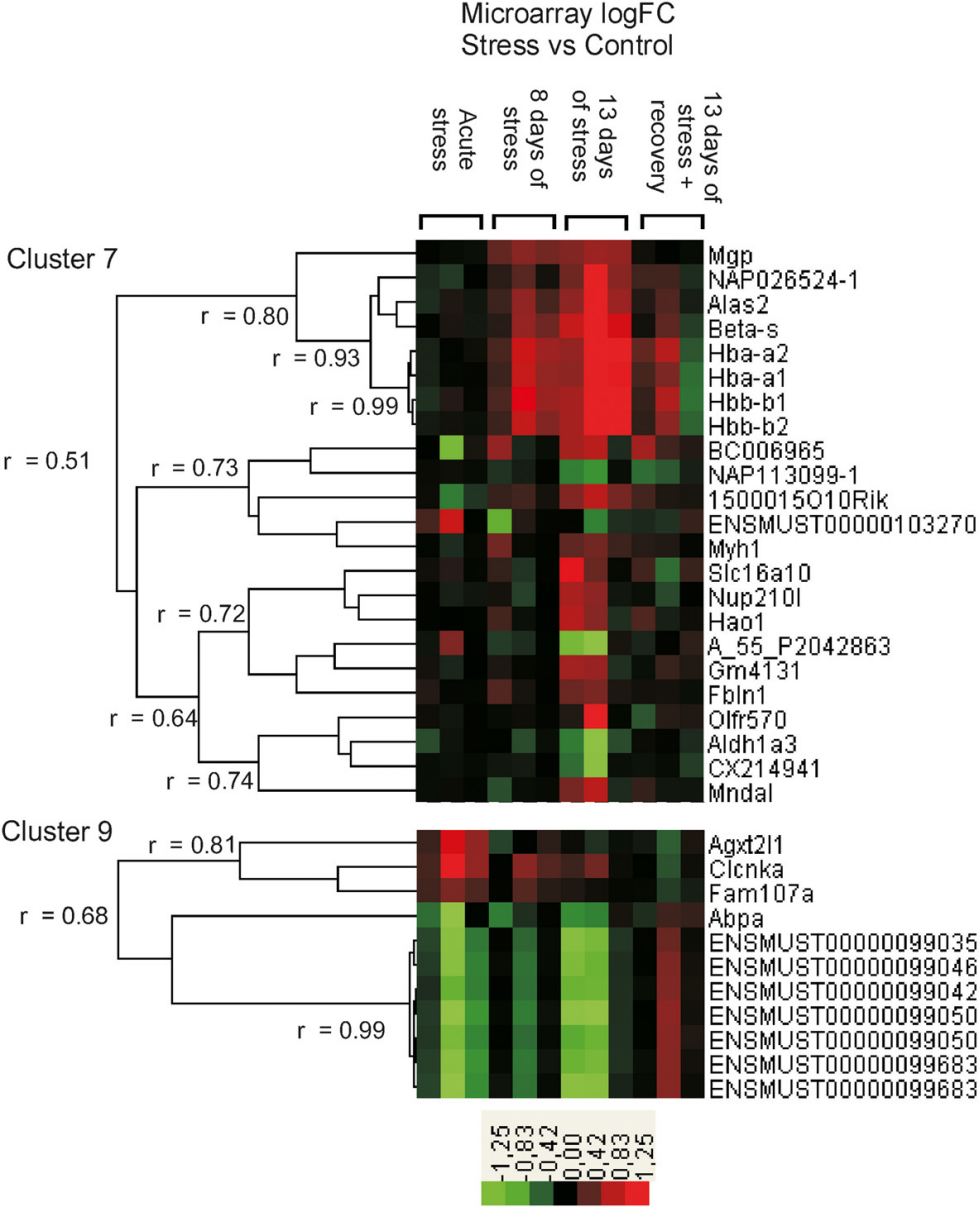
**Expression pattern of genes assigned to cluster 7 and 9.**

**Figure 8:**
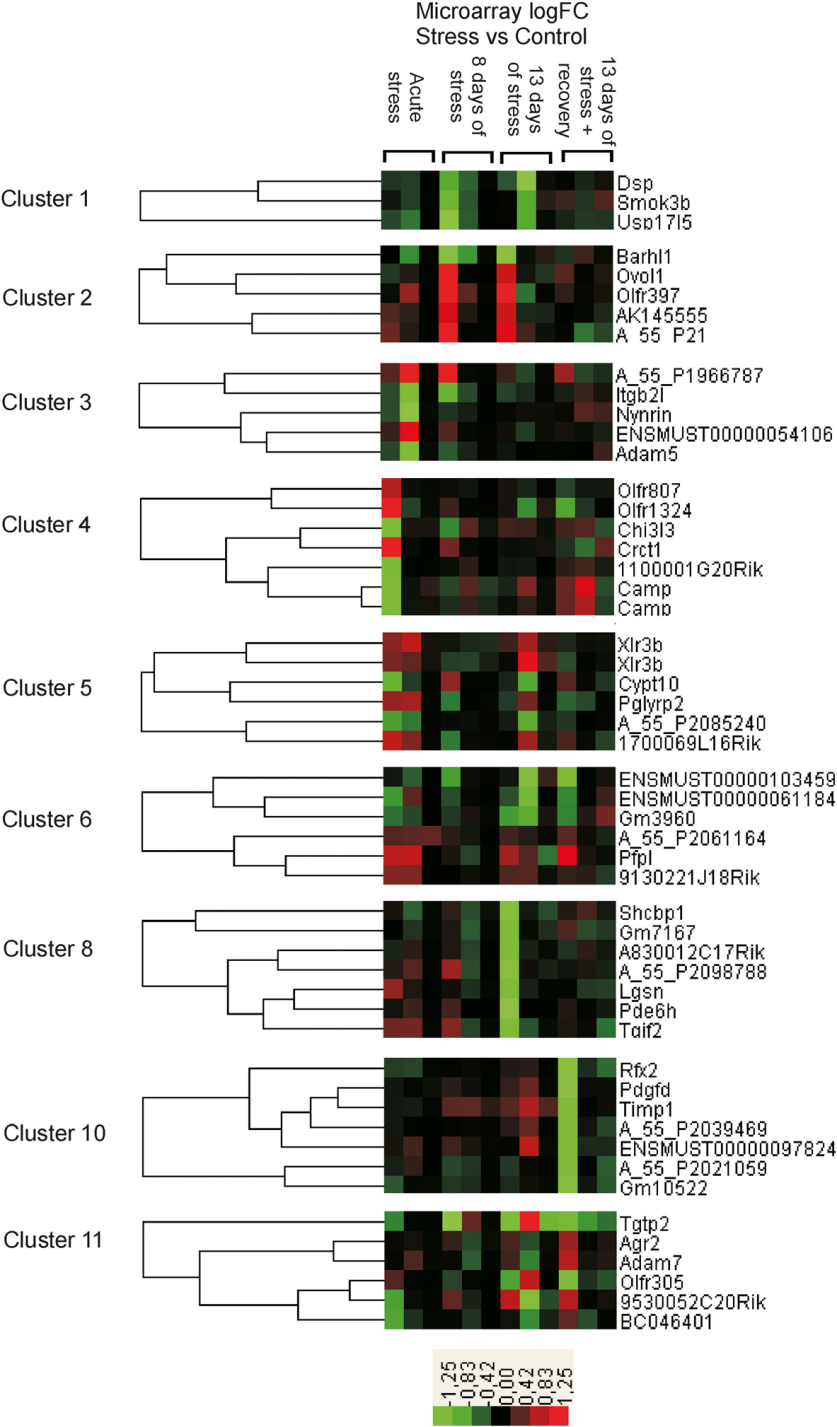
**Expression pattern of genes assigned to cluster 1–6, 8 and 9–11.** Dendrograms show only examples of genes belonging to each cluster with the exception of cluster 1 and 11. For full list of genes see Additional file 1.

The analysis of microarray data revealed significant differences in transcriptomic profiles between stressed and control mice at all of the studied time points (Additional file 1). Although we detected 662 transcripts that were up- or down-regulated by different stress regimes (Table 3), only few genes were significantly regulated at more than one treatment group (Figure 6). The analysis revealed that significantly regulated genes could be grouped into 11 clusters characterized by distinct pattern of expression (Figures 7 and 8; Additional file 1). The most consistent transcriptomic changes that correlated with duration of stress were found in cluster 7 (Figure 7). The core of this cluster contained highly correlated genes coding for hemoglobin (*Hbb-b1*, *Hbb-b2*, *Hba-a1*, *Hba-a2*, *Beta-S*) and two other genes involved in heme synthesis (*Alas2*) and vascular homeostasis (*Mgp*). Expression of these genes was not altered by acute stress, but was progressively increased in animals subjected to 8 and 13 days of stress (Figure 7, Additional file 1). Cluster 7, additionally, contained 14 genes that were up- or down- regulated only after 13 days of stress (Figure 7). Consistent pattern of expression was also found in cluster 9, which contained transcripts of unknown functions that were down-regulated after acute and chronic stress (Figure 7). This cluster contained also 4 other transcripts that were significantly up-regulated (*Agxt2l1*, *Clcnka*, *Fam107a*) or down-regulated (*Abpa*) but only after acute stress (Figure 7). The remaining clusters displayed much less consistent pattern characterized by high variability between the different pools of RNA (Figure 8). In most cases, genes belonging to these clusters were significantly regulated only in one of the stress groups and, frequently, large differences were restricted to one out of three pools from the group. Exceptions were *Timp1* (cluster 10), which was up-regulated in all 3 pools from the chronic stress group (13 days of stress), and *Tgtp2* (cluster 11), which was down-regulated in all 3 pools from the recovery group (Figure 8).

**Table 3:** **Summary of microarray data**

	**Acute stress**	**8 days of stress**	**13 days of stress**	**13 days of stress +5 days of recovery**	**Total**
Up-regulated transcripts	145	70	56	47	318
Down-regulated transcripts	138	48	65	93	344
Total	283	118	121	140	662

### Validation of microarray results

**Figure 9:**
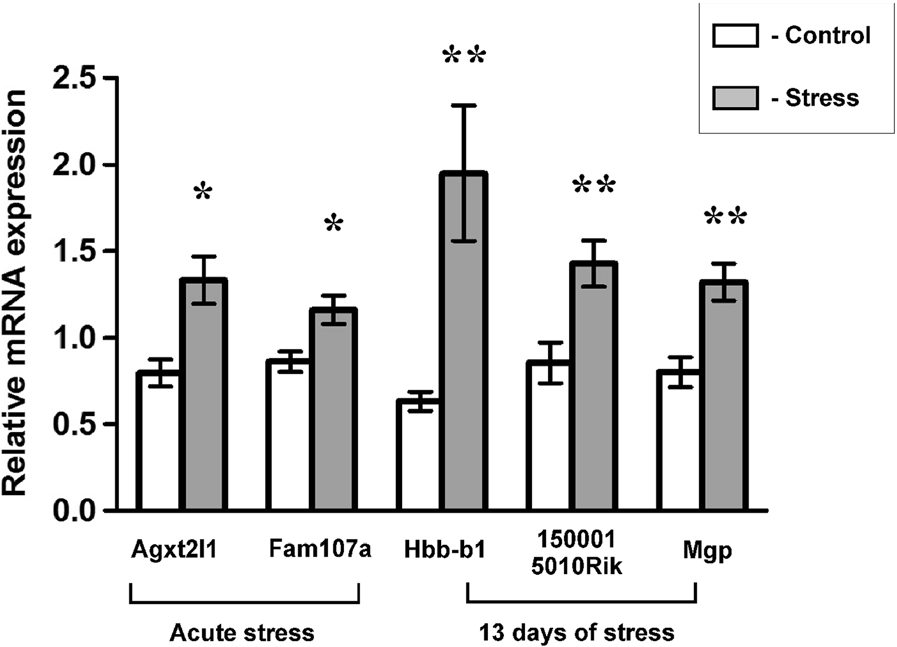
**Quantitative PCR validation of microarray data.** Values are presented as Mean ± SEM. N = 11–12, * - p < 0.05, ** - p < 0.01; compared with corresponding control group.

qPCR confirmed that *Agxt2l1* and *Fam107a* were significantly up-regulated by acute stress, whereas *Hbb-b1*, *Mgp* and *1500015O10Rik* were significantly up-regulated by chronic stress (Figure 9).

### Correlation between organ weights and gene expression

#### Acute stress

**Figure 10:**
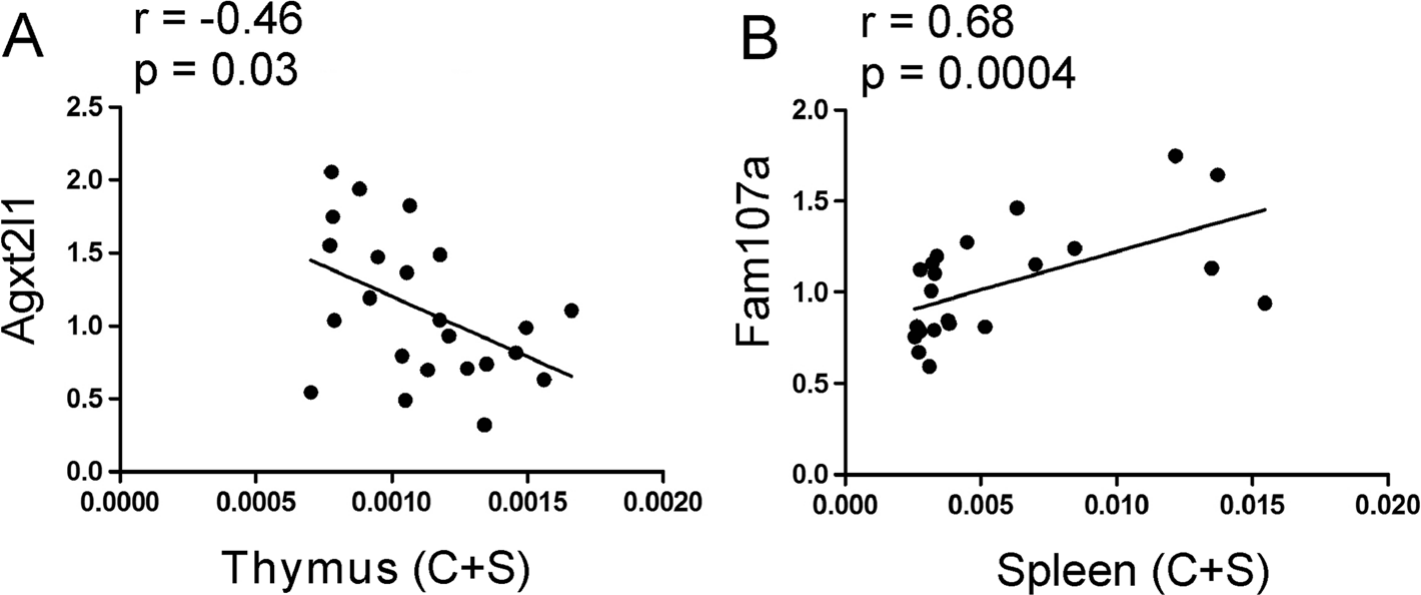
**Correlation between expression of genes affected by acute stress and weight of thymus (A) and spleen (B) calculated per 1 g of body weight.** C + S denotes that presented are data from both control and stressed animals.

Thymic weight was negatively correlated with expression of *Agxt2l1* and this relationship was significant for the pooled data containing results from the control and stressed mice (Figure 10A). Expression of *Fam107a* was positively correlated with weight of spleen and this relationship was significant both in case of pooled data (p < 0.001; Figure 10B) and within control (p < 0.05) and stress group (p < 0.01). Finally, there was also a correlation between weight of thymus and spleen in control group (p < 0.05). Other correlations were not significant (Additional file 2).

#### Chronic stress

**Figure 11:**
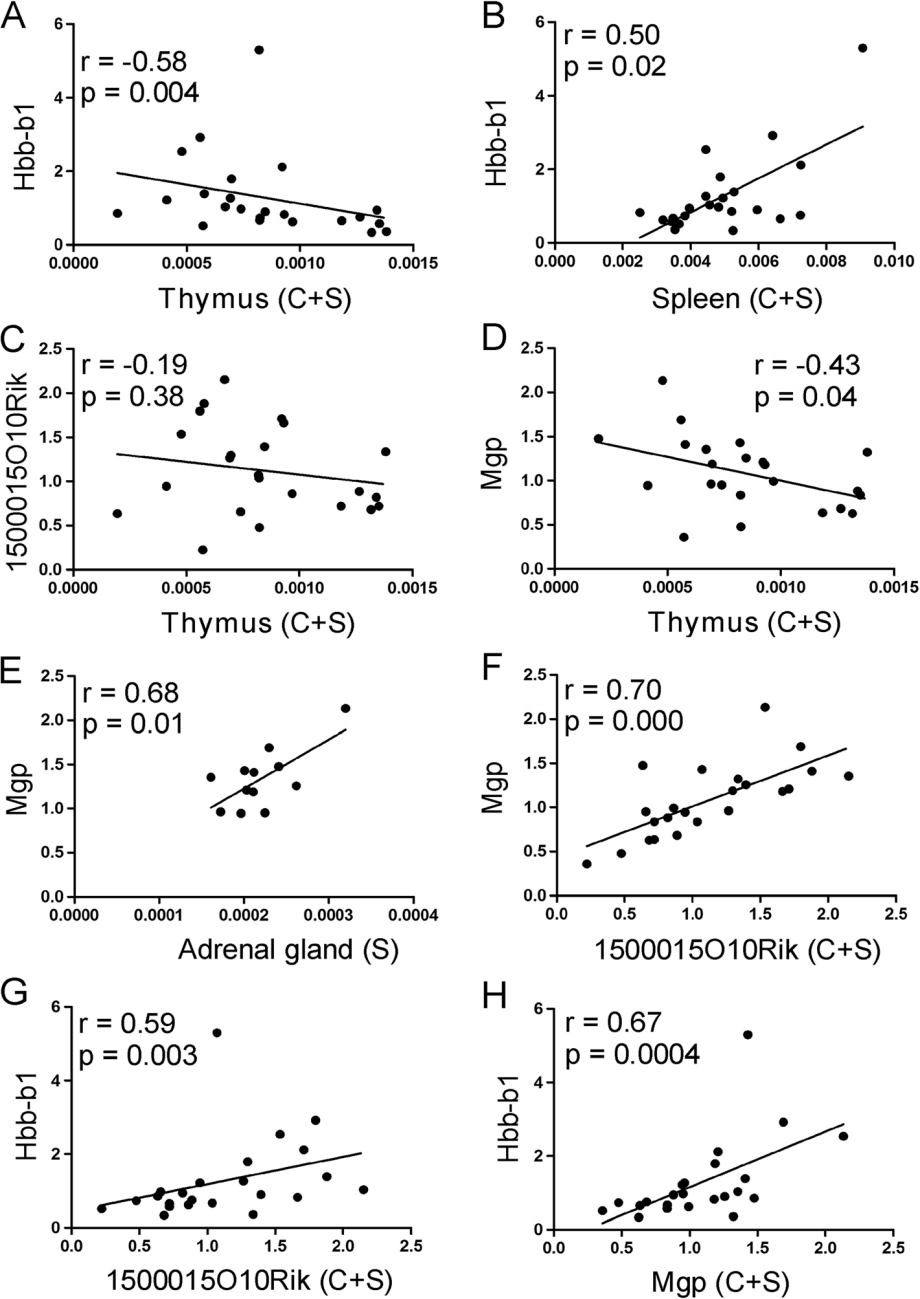
**Correlation between expression of genes affected by chronic stress (13 days) and weight of thymus (A, C, D), spleen (B) and adrenal glands (E) calculated per 1 g of body weight.** Correlation between Mgp, 1500015O10Rik and Hbb-b1 is presented in panel **F**, **G** and **H**. C + S denotes that presented are data from both contron and stressed animals, S denotes correlation only within stress group.

Thymic weight was negatively correlated with expression of *Hbb-b1* and *Mgp* (Figure 11A and D) while weight spleen was positively correlated with expression of *Hbb-b1* (Figure 11B). Weight of adrenal glands correlated positively with expression of *Mgp* but this effect was significant only within the stress group (Figure 11E). There was also a significant correlation between expression of *Hbb-b1*, *Mgp* and *1500015O10Rik* (Figure 11F,G and H). Other correlations were not significant (Additional file 2).

## Discussion

### Indices of stress reactivity

Differences in corticosterone concentration, food intake, body weight gain and size of thymus and spleen confirmed effectiveness of the applied stress paradigm. The observed thymic involution is a classic early symptom of stress that was originally described by Hans Selye [21,36] and since then has been shown repeatedly to be a sensitive and reliable index of stress in rodents [23,37,38]. In our experiment, the degree of thymic involution correlated with the duration of stress and was partly reversed during the recovery period consistently with previously published data [37]. The weight of spleen was less sensitive measure of stress reaction because significant differences were seen only in mice subjected to the longest period of social stress. Previously, it has been shown that splenic enlargement is a typical symptom of chronic social stress in rodents [22,23,39,40]. The observed stress-induced changes in feeding behavior and body weight gain are consistent with the literature data [41]. Decreases in food intake and body weight gain were induced by acute stress and returned to the baseline during the course of experiment. Increased food intake proved also to be sensitive index of recovery processes following chronic social stress.

### Hemoglobin (*Hbb-b1*, *Hbb-b2*, *Hba-a1*, *Hba-a2*, and *Beta-S*) genes

The most important finding of our study was identification of hemoglobin genes as potential markers of chronic social stress in mice. First, expression of *Hbb-b1* was correlated with weights of spleen and thymus that were used as indices of stress reactivity (Figure 11A and B). Second, prefrontal cortex expression of (*Hbb-b1*, *Hbb-b2*, *Hba-a1*, *Hba-a2*, *Beta-S*) was not altered by acute stress, but was progressively increased in animals subjected to 8 and 13 days of repeated social stress (Figure 7, Additional file 1). It is important to note that these transcriptomic changes constitute an independent replication of results because each stress group was compared with its own separate control group. Furthermore, changes in expression of hemoglobin genes were not restricted to prefrontal cortex because similar pattern of expression was found in hippocampal tissue collected from the same mice (Stankiewicz et al., unpublished data). These data are also consistent with the results obtained in tethered pigs that displayed increased expression of hemoglobin beta both in the hippocampus and frontal cortex [16]. Furthermore, the level of hemoglobin expression correlated with the duration of stress both in pigs [16] and mice (present study). Therefore, our mouse model of social stress mirrored transcriptomic indices of stress observed in another species. Comparison of published data shows, however, that this transcriptomic pattern is not consistent across different models of stress in rodents. Partly overlapping results were obtained in the mouse model of chronic mild stress (CMS), which is characterized by successive application of various stressors, such as cage tilting, immobilization, altered lighting cycle, and social encounters. Lisowski et al. [42] reported increased hippocampal level of *Hba-a1* transcript in two different lines of mice subjected to CMS, but these results were not reproduced in prefrontal cortex [43-45]. Opposite changes in hippocampus and amygdala expression of hemoglobin alpha and beta (*Hba-a2* and *Hbb*) were found in rats subjected to chronic restraint stress [46]. However, changes in expression of hemoglobin were not reported in other models of stress, such as repeated forced swimming [47], unavoidable electric shocks [48,49], sub-chronic restraint [50], and chronic inflammatory pain [51]. Relatively low reproducibility of results is not surprising considering recent meta-analysis of microarray experiments investigating pain-induced changes in brain transcriptome [14]. Comparison of data from 20 papers yielded list of 2254 pain-related genes but only seven genes were reported in at least 7 independent studies [14]. In the field of stress research there is a high variability of applied procedures and, therefore, the replicability of stress-induced changes can be even lower than in the case of pain studies.

An important question is physiological mechanism underlying changes in brain expression of hemoglobin. Hemoglobin is expressed at high level in erythrocytes but it is also present in neurons [52-55]. Hemoglobin plays an important role in neuronal respiration, oxidative stress, and response to injury [52-54]. Additionally, neuronal hemoglobin is used by cells to produce hemoglobin-derived peptides (hemorphins, neokyotrophin and hemopressins) acting at opioid and cannabinoid receptors [56]. Increase in frontal expression of hemoglobin genes (*Hba-a1*, *Hba-a2*, *Hbb-b1*) was induced by chronic peripheral inflammation [57] and neuronal expression of *Hba* and *Hbb* genes was increased after intracerebral hemorrhage [53] and ischemia [58]. Up-regulation of *Hba-a1* and *Hbb* has been also found in brains of aged rats [59]. Human HbA2 and HbF were associated with disease severity in bipolar disorder with a likely protective role of HbA2 against post-partum episodes [60]. Therefore, increased level of hemoglobin genes is observed in different pathological states. Unfortunately, we do not know whether the stress-induced changes in expression of hemoglobin genes result from increased expression in neurons, increased blood flow or from increased accumulation of blood in tissue because of changes in local vascular tone, decreased flexibility or occlusion of vessels. However, the fact that expression of hemoglobin was correlated with weight of thymus and spleen suggests that expression of hemoglobin reflected general changes in the state of organism exposed to chronic stress.

### Heme synthesis (*Alas2*) and vascular homeostasis (*Mgp*) genes

The increased expression of hemoglobin genes in prefrontal cortex was associated with increased expression of *Alas2* and *Mgp* in animals subjected to 8 and 13 days of stress (Figure 7, Additional file 1). Expression of *Mgp* was correlated with weight of adrenal glands in stressed animals and with weight of thymus (Figure 11D and E). Prefrontal up-regulation of *Mgp* and *Alas2* was also found in hippocampal tissue collected from the same mice (Stankiewicz et al., unpublished). *Alas2* codes for a key enzyme involved in heme synthesis. Previously, up-regulation of both *Alas2* and hemoglobin genes was found in brain tissue after cerebral artery occlusion [61], prolonged peripheral inflammation [57], in spinal cord after MPTP intoxication [62], and in brain arteriovenous malformations [63]. The same pattern of increased expression of *Alas2, Hba-a1* and *Hbb* was also reported in brains of aged rats [59]. Matrix Gla protein (*Mgp*/*Mglap*) is expressed in bones [64,65], cartilage [65] and vascular smooth muscles [66]. In vascular system MGP plays a homeostatic role in preventing pathological calcification in response to increased level of calcium ions [67]. Increased expression of *Mgp* is associated with different pathological states, such as vascular calcification [68,69], vascular response to renal failure [70], myocardial infarction, and pressure overload [71]. Previously, up-regulation of *Mgp* together with increased level of *Alas2* and hemoglobin genes was found 3 days after cerebral artery occlusion [61] and in cortex of mice with chronic peripheral inflammation [57]. Our experiment showed that animals subjected to the longest period of stress had also increased level of other genes related to vascular system, such as *Fbln1*, *Ppbp* and *Timp1*. Fibulin-1 (*Fbln1*) is a calcium-binding component of the extracellular matrix that surrounds vascular smooth muscle and is involved in thrombosis and platelet adhesion after vascular injury [72-74]. Fibulin-1 was also found to be a component of atherosclerotic lesions [75]. PPBP (pro-platelet basic protein/*Nap-2*) is a chemoattractant that guides leukocytes to sites of vascular injury [76]. The pattern of increased expression of *Ppbp, Alas2* and hemoglobin genes was found previously in spinal cord of mice exposed to toxic effect of MPTP [62]. Expression of *Timp1* (tissue inhibitor of metalloproteinase 1) is increased after focal cerebral ischemia induced by cerebral artery occlusion [61,77] and intracerebral hemorrhage [78]. Furthermore, it has been also showed that ischemia-induced up-regulation of *Timp1* was associated with increased expression of three other aforementioned genes (*Alas2, Hbb-b1 and Mgp*) [61] while hemorrhage induced pattern of increased expression of *Timp1* and human hemoglobin genes (*Hbb*, *Hba1*, *Hba2*) [78]. Experiments performed in knockout mice show that *Timp1* is involved in vascular wound healing [79] and confers protection against blood–brain barrier disruption [77] and progression of vascular pathologies [80].

Elevated brain level of hemoglobin genes mRNA in animals subjected to chronic social stress was also associated with increased level of other genes (*Cntfr, 1500015O10Rik, SLC16A10 and Mndal*) involved in injury and inflammatory responses. *Cntfr* (ciliary neurotrophic factor receptor) has been found previously to be up-regulated after brain injury [81-83] and to protect against neuronal death [84,85]. *1500015O10Rik* (*Ecrg4*) codes for a hormone-like peptide called augurin and is involved in the injury response [86,87] and brain aging [88]. Prefrontal up-regulation of *1500015O10Rik* was also found in mice subjected to chronic mild stress [43]. *SLC16A10* (monocarboxylic acid transporter) has been previously reported to be closely related to cerebral ischemia [89], whereas *Mndal* (myeloid nuclear differentiation antigen-like) is an interferon-inducible gene expressed during inflammation [90]. Likewise, two poorly described genes from cluster 6 (*BC006965* and *Trbv13-2* encoding ENSMUST00000103270) participate in cytokine signaling pathways [91,92].

The observed up-regulation of genes associated with vascular system suggests that stress may affect brain function through the stress-induced dysfunction of the vascular system. It is well known that acute stress triggers sharp increases in blood pressure [93,94] and that chronic stress causes hypertension in genetically predisposed individuals [95,96]. It has been found that acute stress induces damage to vascular endothelium in animal studies [97,98] and triggers endothelial dysfunction in humans [99,100]. Clinical data indicate also that hemodynamic changes caused by hypertension affect cognition because increased blood pressure triggers alterations in cerebral artery structure and function [101,102]. Vascular remodeling, in turn, impairs both blood flow and blood–brain barrier and induces inflammation and oxidative stress [101,102]. Therefore, vascular system may constitute a link between stress and stress-induced brain pathology.

Another important finding was up-and down-regulation of several genes constituting cluster 9. Most of these genes were significantly regulated by acute stress. Perhaps the most interesting is *Fam107a* (*Drr1*) and *Agxt2l1* (*Etnppl*) that are highly expressed in brain [103,104]. Function of *Agxt2l1* is poorly characterized but it is known that *Fam107a* is linked to such crucial processes as long-term potentiation (LTP) and cognition [105]. Increased expression of *Fam107a* was found in hypothalamus, hippocampus and lateral septum after acute glucocorticoid receptor activation and after exposure to various stressors such as maternal deprivation, food deprivation and social defeat [105,106]. Our study showed for the first time that expression of *Fam107a* is also increased in prefrontal cortex after acute social stress. Interestingly, *Fam107a* and *Agxt2l1* are deregulated in prefrontal cortex of patients with schizophrenia and bipolar disorder [107,108]. Moreover, *Agxt2l1* gene expression was changed in mice brain after lithium treatment; a mood stabilizer for bipolar disorder [109]. There is some evidence that psychogenic stress modulates development and severity of psychiatric diseases, such as schizophrenia and bipolar disorder [110-112]. Therefore, these two genes may constitute a link between stress and psychiatric diseases. Another prominent part of cluster 9 is comprised of transcripts *ENSMUST0000009935/-46/-42/-50/-83*, that are products of closely related genes (*Gm10715/10718/10717/10720/10800*, respectively) belonging to family *ENSFM00360000113264* [113]. Function of these predicted protein-coding genes is yet unknown, as are their human homologs. Our findings provide first report of their regulation in context of stress in the brain.

### Methodological consideration

In our study we focused on genes with stable expression across pools of RNA to avoid the effect of biological outliers that can significantly affect results obtained in the pooled samples [114]. Second, we searched for transcriptomic changes that were independently replicated in at least two groups of stressed animals. This approach allowed us to limit large amount of data to several genes that were consistently affected by social encounters. Importantly, expression of these genes was correlated with physiological indices of stress. Microarray analysis yielded also large number of genes characterized by high variability of expression between the pools of RNA. Furthermore, most of genes were not replicated in different groups of stressed animals consistently with previous experimental and review studies [14,115]. Difficulty to generalize most of the obtained results highlights the need for focusing on replicability of transcriptomic changes detected by microarrays.

## Conclusions

The most important finding is identification of hemoglobin genes as potential markers of chronic social stress in mice (*Hbb-b1, Hbb-b2, Hba-a1, Hba-a2, Beta-S*). Up-regulation of genes associated with injury, inflammation and vascular system suggests that social stress may affect brain function through the stress-induced dysfunction of the vascular system. This data are consistent with recent finding that repeated social defeat promotes migration of peripheral macrophages to the brain [39]. Furthermore, there is an increasing interest in function of brain hemoglobin [52-55] and some other genes such as *1500015O10Rik* that codes for a hormone-like peptide called augurin [86,88]. Finally, we report stress-induced changes in expression of genes involved in psychiatric diseases such as *Fam107a* and *Agxt2l1*. Therefore, the observed transcriptomic changes may constitute a link between stress and mental health.

## Additional files

Additional file 1:
**Complete list of genes that were significantly up - or down-regulated by social stress.**
Additional file 2:
**Correlation between selected genes and weights of internal organs.**

## References

[1] Snyder BK, Roghmann KJ, Sigal LH (1993). Stress and psychosocial factors: effects on primary cellular immune response. J Behav Med.

[2] Lee DJ, Meehan RT, Robinson C, Smith ML, Mabry TR (1995). Psychosocial correlates of immune responsiveness and illness episodes in US Air Force Academy cadets undergoing basic cadet training. J Psychosom Res.

[3] Glaser R, Pearson GR, Bonneau RH, Esterling BA, Atkinson C, Kiecolt-Glaser JK (1993). Stress and the memory T-cell response to the Epstein-Barr virus in healthy medical students. Health Psychol.

[4] Weinberg A, Creed F (2000). Stress and psychiatric disorder in healthcare professionals and hospital staff. Lancet.

[5] Dunn AJ, Swiergiel AH (2008). The role of corticotropin-releasing factor and noradrenaline in stress-related responses, and the inter-relationships between the two systems. Eur J Pharmacol.

[6] Pickering TG (2001). Mental stress as a causal factor in the development of hypertension and cardiovascular disease. Curr Hypertens Rep.

[7] Rozanski A, Blumenthal JA, Kaplan J (1999). Impact of psychological factors on the pathogenesis of cardiovascular disease and implications for therapy. Circulation.

[8] Courtin J, Chaudun F, Rozeske RR, Karalis N, Gonzalez-Campo C, Wurtz H, Abdi A, Baufreton J, Bienvenu TC, Herry C (2014). Prefrontal parvalbumin interneurons shape neuronal activity to drive fear expression. Nature.

[9] Likhtik E, Stujenske JM, Topiwala MA, Harris AZ, Gordon JA (2014). Prefrontal entrainment of amygdala activity signals safety in learned fear and innate anxiety. Nat Neurosci.

[10] Bourne AR, Mohan G, Stone MF, Pham MQ, Schultz CR, Meyerhoff JL, Lumley LA (2013). Olfactory cues increase avoidance behavior and induce Fos expression in the amygdala, hippocampus and prefrontal cortex of socially defeated mice. Behav Brain Res.

[11] Singh R (2013). Signal oscillation is another reason for variability in microarray-based gene expression quantification. PLoS One.

[12] Shippy R, Sendera TJ, Lockner R, Palaniappan C, Kaysser-Kranich T, Watts G, Alsobrook J (2004). Performance evaluation of commercial short-oligonucleotide microarrays and the impact of noise in making cross-platform correlations. BMC Genomics.

[13] Scherer A, Dai M, Meng F (2013). Impact of experimental noise and annotation imprecision on data quality in microarray experiments. Methods Mol Biol.

[14] LaCroix-Fralish ML, Austin JS, Zheng FY, Levitin DJ, Mogil JS (2011). Patterns of pain: meta-analysis of microarray studies of pain. Pain.

[15] Klebanov L, Qiu X, Welle S, Yakovlev A (2007). Statistical methods and microarray data. Nat Biotechnol.

[16] van der Staay FJ, Schuurman T, Hulst M, Smits M, Prickaerts J, Kenis G, Korte SM (2010). Effects of chronic stress: a comparison between tethered and loose sows. Physiol Behav.

[17] Li XH, Chen JX, Yue GX, Liu YY, Zhao X, Guo XL, Liu Q, Jiang YM, Bai MH (2013). Gene expression profile of the hippocampus of rats subjected to chronic immobilization stress. PLoS One.

[18] Swiergiel AH, Smagin GN, Johnson LJ, Dunn AJ (1997). The role of cytokines in the behavioral responses to endotoxin and influenza virus infection in mice: effects of acute and chronic administration of the interleukin-1-receptor antagonist (IL-1ra). Brain Res.

[19] Litvin Y, Blanchard DC, Pentkowski NS, Blanchard RJ (2007). A pinch or a lesion: a reconceptualization of biting consequences in mice. Aggress Behav.

[20] Kudryavtseva NN, Bondar NP, Alekseyenko OV (2000). Behavioral correlates of learned aggression in male mice. Aggress Behav.

[21] Selye H (1936). A syndrome produced by diverse nocuous agents. Nature.

[22] Engler H, Bailey MT, Engler A, Stiner-Jones LM, Quan N, Sheridan JF (2008). Interleukin-1 receptor type 1-deficient mice fail to develop social stress-associated glucocorticoid resistance in the spleen. Psychoneuroendocrinology.

[23] Blanchard DC, Sakai RR, McEwen B, Weiss SM, Blanchard RJ (1993). Subordination stress: behavioral, brain, and neuroendocrine correlates. Behav Brain Res.

[24] Ling S, Jamali F (2003). Effect of cannulation surgery and restraint stress on the plasma corticosterone concentration in the rat: application of an improved corticosterone HPLC assay. J Pharm Pharm Sci.

[25] Paxinos G, Franklin KBJ (2001). The Mouse Brain in Stereotaxic Coordinates.

[26] Pfaffl MW (2001). A new mathematical model for relative quantification in real-time RT-PCR. Nucleic Acids Res.

[27] Hancock AA, Bush EN, Stanisic D, Kyncl JJ, Lin CT (1988). Data normalization before statistical analysis: keeping the horse before the cart. Trends Pharmacol Sci.

[28] Yuan JS, Reed A, Chen F, Stewart CN (2006). Statistical analysis of real-time PCR data. BMC Bioinformatics.

[29] Datson NA, Speksnijder N, Mayer JL, Steenbergen PJ, Korobko O, Goeman J, de Kloet ER, Joels M, Lucassen PJ (2012). The transcriptional response to chronic stress and glucocorticoid receptor blockade in the hippocampal dentate gyrus. Hippocampus.

[30] Smyth GK, Gentleman R, Carey V, Dudoit S, Irizarry R, Huber W (2005). Limma: Linear Models for Microarray Data. Bioinformatics and Computational Biology Solutions Using R and Bioconductor.

[31] Ritchie ME, Silver J, Oshlack A, Holmes M, Diyagama D, Holloway A, Smyth GK (2007). A comparison of background correction methods for two-colour microarrays. Bioinformatics.

[32] Smyth GK, Speed T (2003). Normalization of cDNA microarray data. Methods.

[33] Benjamini Y, Hochberg Y (1995). Controlling the false discovery rate: a practical and powerful approach to multiple testing. J Royal Stat Soc B.

[34] Saldanha AJ (2004). Java treeview–extensible visualization of microarray data. Bioinformatics.

[35] Michener CD, Sokal RR (1957). A quantitative approach to a problem of classification. Evolution.

[36] Selye H (1936). Thymus and adrenals in the response of the organism to injuries and intoxications. Br J Exp Pathol.

[37] Dominguez-Gerpe L, Rey-Mendez M (1997). Time-course of the murine lymphoid tissue involution during and following stressor exposure. Life Sci.

[38] Pertsov SS (2006). Effect of melatonin on the thymus, adrenal glands, and spleen in rats during acute stress. Bull Exp Biol Med.

[39] Wohleb ES, Powell ND, Godbout JP, Sheridan JF (2013). Stress-induced recruitment of bone marrow-derived monocytes to the brain promotes anxiety-like behavior. J Neurosci.

[40] Hanke ML, Powell ND, Stiner LM, Bailey MT, Sheridan JF (2012). Beta adrenergic blockade decreases the immunomodulatory effects of social disruption stress. Brain Behav Immun.

[41] Maniam J, Morris MJ (2012). The link between stress and feeding behaviour. Neuropharmacology.

[42] Lisowski P, Juszczak GR, Goscik J, Wieczorek M, Zwierzchowski L, Swiergiel AH (2011). Effect of chronic mild stress on hippocampal transcriptome in mice selected for high and low stress-induced analgesia and displaying different emotional behaviors. Eur Neuropsychopharmacol.

[43] Lisowski P, Wieczorek M, Goscik J, Juszczak GR, Stankiewicz AM, Zwierzchowski L, Swiergiel AH (2013). Effects of chronic stress on prefrontal cortex transcriptome in mice displaying different genetic backgrounds. J Mol Neurosci.

[44] Tordera RM, Garcia-Garcia AL, Elizalde N, Segura V, Aso E, Venzala E, Ramirez MJ, Del Rio J (2011). Chronic stress and impaired glutamate function elicit a depressive-like phenotype and common changes in gene expression in the mouse frontal cortex. Eur Neuropsychopharmacol.

[45] Orsetti M, Di Brisco F, Canonico PL, Genazzani AA, Ghi P (2008). Gene regulation in the frontal cortex of rats exposed to the chronic mild stress paradigm, an animal model of human depression. Eur J Neurosci.

[46] Andrus BM, Blizinsky K, Vedell PT, Dennis K, Shukla PK, Schaffer DJ, Radulovic J, Churchill GA, Redei EE (2012). Gene expression patterns in the hippocampus and amygdala of endogenous depression and chronic stress models. Mol Psychiatry.

[47] Aso E, Ozaita A, Serra MA, Maldonado R (2011). Genes differentially expressed in CB1 knockout mice: involvement in the depressive-like phenotype. Eur Neuropsychopharmacol.

[48] Benatti C, Valensisi C, Blom JM, Alboni S, Montanari C, Ferrari F, Tagliafico E, Mendlewicz J, Brunello N, Tascedda F (2012). Transcriptional profiles underlying vulnerability and resilience in rats exposed to an acute unavoidable stress. J Neurosci Res.

[49] Mingmalairak S, Tohda M, Murakami Y, Matsumoto K (2010). Possible involvement of signal transducers and activators of transcription 3 system on depression in the model mice brain. Biol Pharm Bull.

[50] Barreto RA, Walker FR, Dunkley PR, Day TA, Smith DW (2012). Fluoxetine prevents development of an early stress-related molecular signature in the rat infralimbic medial prefrontal cortex. Implications for depression?. BMC Neurosci.

[51] Poh KW, Yeo JF, Stohler CS, Ong WY (2012). Comprehensive gene expression profiling in the prefrontal cortex links immune activation and neutrophil infiltration to antinociception. J Neurosci.

[52] Richter F, Meurers BH, Zhu C, Medvedeva VP, Chesselet MF (2009). Neurons express hemoglobin alpha- and beta-chains in rat and human brains. J Comp Neurol.

[53] He Y, Hua Y, Lee JY, Liu W, Keep RF, Wang MM, Xi G (2010). Brain alpha- and beta-globin expression after intracerebral hemorrhage. Transl Stroke Res.

[54] Schelshorn DW, Schneider A, Kuschinsky W, Weber D, Kruger C, Dittgen T, Burgers HF, Sabouri F, Gassler N, Bach A, Maurer MH (2009). Expression of hemoglobin in rodent neurons. J Cereb Blood Flow Metab.

[55] Biagioli M, Pinto M, Cesselli D, Zaninello M, Lazarevic D, Roncaglia P, Simone R, Vlachouli C, Plessy C, Bertin N, Beltrami A, Kobayashi K, Gallo V, Santoro C, Ferrer I, Rivella S, Beltrami CA, Carninci P, Raviola E, Gustincich S (2009). Unexpected expression of alpha- and beta-globin in mesencephalic dopaminergic neurons and glial cells. Proc Natl Acad Sci U S A.

[56] Gelman JS, Sironi J, Castro LM, Ferro ES, Fricker LD (2010). Hemopressins and other hemoglobin-derived peptides in mouse brain: comparison between brain, blood, and heart peptidome and regulation in Cpefat/fat mice. J Neurochem.

[57] Sarlus H, Wang X, Cedazo-Minguez A, Schultzberg M, Oprica M (2013). Chronic airway-induced allergy in mice modifies gene expression in the brain toward insulin resistance and inflammatory responses. J Neuroinflammation.

[58] He Y, Hua Y, Liu W, Hu H, Keep RF, Xi G (2009). Effects of cerebral ischemia on neuronal hemoglobin. J Cereb Blood Flow Metab.

[59] Burger C, Lopez MC, Baker HV, Mandel RJ, Muzyczka N (2008). Genome-wide analysis of aging and learning-related genes in the hippocampal dentate gyrus. Neurobiol Learn Mem.

[60] Ince B, Guloksuz S, Altinbas K, Oral ET, Alpkan LR, Altinoz MA (2013). Minor hemoglobins HbA2 and HbF associate with disease severity in bipolar disorder with a likely protective role of HbA2 against post-partum episodes. J Affect Disord.

[61] Ramos-Cejudo J, Gutierrez-Fernandez M, Rodriguez-Frutos B, Exposito Alcaide M, Sanchez-Cabo F, Dopazo A, Diez-Tejedor E (2012). Spatial and temporal gene expression differences in core and periinfarct areas in experimental stroke: a microarray analysis. PLoS One.

[62] Choi YG, Yeo S, Hong YM, Kim SH, Lim S (2011). Changes of gene expression profiles in the cervical spinal cord by acupuncture in an MPTP-intoxicated mouse model: microarray analysis. Gene.

[63] Takagi Y, Aoki T, Takahashi JC, Yoshida K, Ishii A, Arakawa Y, Kikuchi T, Funaki T, Miyamoto S (2014). Differential gene expression in relation to the clinical characteristics of human brain arteriovenous malformations. Neurol Med Chir (Tokyo).

[64] Price PA, Urist MR, Otawara Y (1983). Matrix Gla protein, a new gamma-carboxyglutamic acid-containing protein which is associated with the organic matrix of bone. Biochem Biophys Res Commun.

[65] Hale JE, Fraser JD, Price PA (1988). The identification of matrix Gla protein in cartilage. J Biol Chem.

[66] Wallin R, Cain D, Sane DC (1999). Matrix Gla protein synthesis and gamma-carboxylation in the aortic vessel wall and proliferating vascular smooth muscle cells–a cell system which resembles the system in bone cells. Thromb Haemost.

[67] Farzaneh-Far A, Proudfoot D, Weissberg PL, Shanahan CM (2000). Matrix gla protein is regulated by a mechanism functionally related to the calcium-sensing receptor. Biochem Biophys Res Commun.

[68] Shanahan CM, Cary NR, Metcalfe JC, Weissberg PL (1994). High expression of genes for calcification-regulating proteins in human atherosclerotic plaques. J Clin Invest.

[69] Proudfoot D, Skepper JN, Shanahan CM, Weissberg PL (1998). Calcification of human vascular cells in vitro is correlated with high levels of matrix Gla protein and low levels of osteopontin expression. Arterioscler Thromb Vasc Biol.

[70] Lomashvili KA, Wang X, Wallin R, O’Neill WC (2011). Matrix Gla protein metabolism in vascular smooth muscle and role in uremic vascular calcification. J Biol Chem.

[71] Mustonen E, Pohjolainen V, Aro J, Pikkarainen S, Leskinen H, Ruskoaho H, Rysa J (2009). Upregulation of cardiac matrix Gla protein expression in response to hypertrophic stimuli. Blood Press.

[72] Godyna S, Diaz-Ricart M, Argraves WS (1996). Fibulin-1 mediates platelet adhesion via a bridge of fibrinogen. Blood.

[73] Tran H, Tanaka A, Litvinovich SV, Medved LV, Haudenschild CC, Argraves WS (1995). The interaction of fibulin-1 with fibrinogen. A potential role in hemostasis and thrombosis. J Biol Chem.

[74] Tran H, VanDusen WJ, Argraves WS (1997). The self-association and fibronectin-binding sites of fibulin-1 map to calcium-binding epidermal growth factor-like domains. J Biol Chem.

[75] Argraves WS, Tanaka A, Smith EP, Twal WO, Argraves KM, Fan D, Haudenschild CC (2009). Fibulin-1 and fibrinogen in human atherosclerotic lesions. Histochem Cell Biol.

[76] Ghasemzadeh M, Kaplan ZS, Alwis I, Schoenwaelder SM, Ashworth KJ, Westein E, Hosseini E, Salem HH, Slattery R, McColl SR, Hickey MJ, Ruggeri ZM, Yuan Y, Jackson SP (2013). The CXCR1/2 ligand NAP-2 promotes directed intravascular leukocyte migration through platelet thrombi. Blood.

[77] Fujimoto M, Takagi Y, Aoki T, Hayase M, Marumo T, Gomi M, Nishimura M, Kataoka H, Hashimoto N, Nozaki K (2008). Tissue inhibitor of metalloproteinases protect blood–brain barrier disruption in focal cerebral ischemia. J Cereb Blood Flow Metab.

[78] Rosell A, Vilalta A, Garcia-Berrocoso T, Fernandez-Cadenas I, Domingues-Montanari S, Cuadrado E, Delgado P, Ribo M, Martinez-Saez E, Ortega-Aznar A, Montaner J (2011). Brain perihematoma genomic profile following spontaneous human intracerebral hemorrhage. PLoS One.

[79] Lijnen HR, Soloway P, Collen D (1999). Tissue inhibitor of matrix metalloproteinases-1 impairs arterial neointima formation after vascular injury in mice. Circ Res.

[80] Aoki T, Kataoka H, Moriwaki T, Nozaki K, Hashimoto N (2007). Role of TIMP-1 and TIMP-2 in the progression of cerebral aneurysms. Stroke.

[81] Yokota H, Yoshikawa M, Hirabayashi H, Nakase H, Uranishi R, Nishimura F, Sugie Y, Ishizaka S, Sakaki T (2005). Expression of ciliary neurotrophic factor (CNTF), CNTF receptor alpha (CNTFR-alpha) following experimental intracerebral hemorrhage in rats. Neurosci Lett.

[82] Choi JS, Kim SY, Park HJ, Cha JH, Choi YS, Chung JW, Chun MH, Lee MY (2004). Differential regulation of ciliary neurotrophic factor and its receptor in the rat hippocampus in response to kainic acid-induced excitotoxicity. Mol Cells.

[83] Miotke JA, MacLennan AJ, Meyer RL (2007). Immunohistochemical localization of CNTFRalpha in adult mouse retina and optic nerve following intraorbital nerve crush: evidence for the axonal loss of a trophic factor receptor after injury. J Comp Neurol.

[84] Ozog MA, Modha G, Church J, Reilly R, Naus CC (2008). Co-administration of ciliary neurotrophic factor with its soluble receptor protects against neuronal death and enhances neurite outgrowth. J Biol Chem.

[85] Lee N, Robitz R, Zurbrugg RJ, Karpman AM, Mahler AM, Cronier SA, Vesey R, Spearry RP, Zolotukhin S, Maclennan AJ (2008). Conditional, genetic disruption of ciliary neurotrophic factor receptors reveals a role in adult motor neuron survival. Eur J Neurosci.

[86] Podvin S, Gonzalez AM, Miller MC, Dang X, Botfield H, Donahue JE, Kurabi A, Boissaud-Cooke M, Rossi R, Leadbeater WE, Johanson CE, Coimbra R, Stopa EG, Eliceiri BP, Baird A (2011). Esophageal cancer related gene-4 is a choroid plexus-derived injury response gene: evidence for a biphasic response in early and late brain injury. PLoS One.

[87] Shaterian A, Kao S, Chen L, DiPietro LA, Coimbra R, Eliceiri BP, Baird A (2013). The candidate tumor suppressor gene Ecrg4 as a wound terminating factor in cutaneous injury. Arch Dermatol Res.

[88] Kujuro Y, Suzuki N, Kondo T (2010). Esophageal cancer-related gene 4 is a secreted inducer of cell senescence expressed by aged CNS precursor cells. Proc Natl Acad Sci U S A.

[89] Gao J, Yang H, Chen J, Fang J, Chen C, Liang R, Yang G, Wu H, Wu C, Li S (2013). Analysis of serum metabolites for the discovery of amino acid biomarkers and the effect of galangin on cerebral ischemia. Mol Biosyst.

[90] Zhang K, Kagan D, DuBois W, Robinson R, Bliskovsky V, Vass WC, Zhang S, Mock BA (2009). Mndal, a new interferon-inducible family member, is highly polymorphic, suppresses cell growth, and may modify plasmacytoma susceptibility. Blood.

[91] Kass DJ, Yu G, Loh KS, Savir A, Borczuk A, Kahloon R, Juan-Guardela B, Deiuliis G, Tedrow J, Choi J, Richards T, Kaminski N, Greenberg SM (2012). Cytokine-like factor 1 gene expression is enriched in idiopathic pulmonary fibrosis and drives the accumulation of CD4+ T cells in murine lungs: evidence for an antifibrotic role in bleomycin injury. Am J Pathol.

[92] Jia J, Dai M, Zhuang Y (2008). E proteins are required to activate germline transcription of the TCR Vbeta8.2 gene. Eur J Immunol.

[93] Vianna DM, Carrive P (2010). Cardiovascular and behavioural responses to conditioned fear and restraint are not affected by retrograde lesions of A5 and C1 bulbospinal neurons. Neuroscience.

[94] Busnardo C, Tavares RF, Resstel LB, Elias LL, Correa FM (2010). Paraventricular nucleus modulates autonomic and neuroendocrine responses to acute restraint stress in rats. Auton Neurosci.

[95] Bernatowa I, Csizmadiova Z, Kopincova J, Puzserova A (2007). Vascular function and nitric oxide production in chronic social-stress-exposed rats with various family history of hypertension. J Physiol Pharmacol.

[96] Snieder H, Harshfield GA, Barbeau P, Pollock DM, Pollock JS, Treiber FA (2002). Dissecting the genetic architecture of the cardiovascular and renal stress response. Biol Psychol.

[97] Skantze HB, Kaplan J, Pettersson K, Manuck S, Blomqvist N, Kyes R, Williams K, Bondjers G (1998). Psychosocial stress causes endothelial injury in cynomolgus monkeys via beta1-adrenoceptor activation. Atherosclerosis.

[98] Jezova D, Kristova V, Slamova J, Mlynarik M, Pirnik Z, Kiss A, Kriska M (2003). Stress-induced rise in endothelaemia, von Willebrand factor and hypothalamic-pituitary-adrenocortical axis activation is reduced by pretreatment with pentoxifylline. J Physiol Pharmacol.

[99] Sherwood A, Johnson K, Blumenthal JA, Hinderliter AL (1999). Endothelial function and hemodynamic responses during mental stress. Psychosom Med.

[100] Ghiadoni L, Donald AE, Cropley M, Mullen MJ, Oakley G, Taylor M, O’Connor G, Betteridge J, Klein N, Steptoe A, Deanfield JE (2000). Mental stress induces transient endothelial dysfunction in humans. Circulation.

[101] Bink DI, Ritz K, Aronica E, van der Weerd L, Daemen MJ (2013). Mouse models to study the effect of cardiovascular risk factors on brain structure and cognition. J Cereb Blood Flow Metab.

[102] Pires PW, Dams Ramos CM, Matin N, Dorrance AM (2013). The effects of hypertension on the cerebral circulation. Am J Physiol Heart Circ Physiol.

[103] Liebl C, Panhuysen M, Putz B, Trumbach D, Wurst W, Deussing JM, Muller MB, Schmidt MV (2009). Gene expression profiling following maternal deprivation: involvement of the brain renin-angiotensin system. Front Mol Neurosci.

[104] Veiga-da-Cunha M, Hadi F, Balligand T, Stroobant V, Van Schaftingen E (2012). Molecular identification of hydroxylysine kinase and of ammoniophospholyases acting on 5-phosphohydroxy-L-lysine and phosphoethanolamine. J Biol Chem.

[105] Schmidt MV, Schulke JP, Liebl C, Stiess M, Avrabos C, Bock J, Wochnik GM, Davies HA, Zimmermann N, Scharf SH, Trumbach D, Wurst W, Zieglgansberger W, Turck C, Holsboer F, Stewart MG, Bradke F, Eder M, Muller MB, Rein T (2011). Tumor suppressor down-regulated in renal cell carcinoma 1 (DRR1) is a stress-induced actin bundling factor that modulates synaptic efficacy and cognition. Proc Natl Acad Sci U S A.

[106] Masana M, Su YA, Liebl C, Wang XD, Jansen L, Westerholz S, Wagner KV, Labermaier C, Scharf SH, Santarelli S, Hartmann J, Schmidt MV, Rein T, Muller MB (2014). The stress-inducible actin-interacting protein DRR1 shapes social behavior. Psychoneuroendocrinology.

[107] Kim S, Choi KH, Baykiz AF, Gershenfeld HK (2007). Suicide candidate genes associated with bipolar disorder and schizophrenia: an exploratory gene expression profiling analysis of post-mortem prefrontal cortex. BMC Genomics.

[108] Shao L, Vawter MP (2008). Shared gene expression alterations in schizophrenia and bipolar disorder. Biol Psychiatry.

[109] McQuillin A, Rizig M, Gurling HM (2007). A microarray gene expression study of the molecular pharmacology of lithium carbonate on mouse brain mRNA to understand the neurobiology of mood stabilization and treatment of bipolar affective disorder. Pharmacogenet Genomics.

[110] Carr CP, Martins CM, Stingel AM, Lemgruber VB, Juruena MF (2013). The role of early life stress in adult psychiatric disorders: a systematic review according to childhood trauma subtypes. J Nerv Ment Dis.

[111] Lederbogen F, Haddad L, Meyer-Lindenberg A (2013). Urban social stress–risk factor for mental disorders. The case of schizophrenia. Environ Pollut.

[112] Bratlien U, Oie M, Haug E, Moller P, Andreassen OA, Lien L, Melle I (2014). Environmental factors during adolescence associated with later development of psychotic disorders - a nested case–control study. Psychiatry Res.

[113] Flicek P, Ahmed I, Amode MR, Barrell D, Beal K, Brent S, Carvalho-Silva D, Clapham P, Coates G, Fairley S, Fitzgerald S, Gil L, Garcia-Giron C, Gordon L, Hourlier T, Hunt S, Juettemann T, Kahari AK, Keenan S, Komorowska M, Kulesha E, Longden I, Maurel T, McLaren WM, Muffato M, Nag R, Overduin B, Pignatelli M, Pritchard B, Pritchard E (2013). Ensembl 2013. Nucleic Acids Res.

[114] Paulsen SJ, Larsen LK, Jelsing J, Janssen U, Gerstmayer B, Vrang N (2009). Gene expression profiling of individual hypothalamic nuclei from single animals using laser capture microdissection and microarrays. J Neurosci Methods.

[115] Lauridsen JB, Johansen JL, Rekling JC, Thirstrup K, Moerk A, Sager TN (2011). Regulation of the Bcas1 and Baiap3 transcripts in the subthalamic nucleus in mice recovering from MPTP toxicity. Neurosci Res.

