# Supplementary material for: Social stress increases expression of hemoglobin genes in mouse prefrontal cortex
Source: BMC Neurosci. 2014 Dec 4;15:130. doi: 10.1186/s12868-014-0130-6 (PMC4269175; doi:10.1186/s12868-014-0130-6)
Supplement: Additional file 2: — Correlation between selected genes and weights of internal organs. [file 12868_2014_130_MOESM2_ESM.pdf]

Correlation between data (acute stress)

	Thymus			Spleen			Arenal gland			Agxt2l1		
	All animals	Control group	Stress group	All animals	Control group	Stress group	All animals	Control group	Stress group	All animals	Control group	Stress group
Thymus	---			---			---------------------------------------			---------------------------------------		
Spleen	r = 0.11<br>p = 0.60	r = 0.60<br>p = **0.04**	r = -0.21<br>p = 0.51									
Adrenal gland	r = 0.27<br>p = 0.20	r = 0.30<br>p = 0.35	r = 0.05<br>p = 0.88	r = 0.26<br>p = 0.22	r = 0.47<br>p = 0.12	r = 0.31<br>p = 0.32						
Agxt2l1	r = -0.46<br>p = **0.03**	r = -0.10<br>p = 0.76	r = -0.20<br>p = 0.53	r = -0.07<br>p = 0.76	r = -0.38<br>p = 0.25	r = -0.10<br>p = 0.75	r = -0.38<br>p = 0.08	r = -0.58<br>p = 0.06	r = -0.17<br>p =0.59			
Fam107a	r = -0.31<br>p = 0.16	r = 0.31<br>p = 0.36	r = -0.17<br>p = 0.59	r = 0.68<br>p = **0.0004**	r = 0.66<br>p = **0.03**	r = 0.77<br>p = **0.003**	r = -0.09<br>p = 0.69	r = 0.01<br>p = 0.98	r = 0.10<br>p = 0.75	r = 0.21<br>p = 0.33	r = -0.17<br>p = 0.61	r = -0.06<br>p = 0.85

Correlation between data (13 days of stress)

	Thymus			Spleen			Arenal gland		
	All animals	Control group	Stress group	All animals	Control group	Stress group	All animals	Control group	Stress group
Thymus	---			---			---------------------------------------		
Spleen	r = -0.07<br>p = 0.73	r = 0.24<br>p = 0.46	r = 0.35<br>p = 0.27						
Adrenal gland	r = -0.34<br>p = 0.11	r = -0.05<br>p = 0.89	r = -0.26<br>p = 0.42	r = 0.20<br>p = 0.34	r = 0.10<br>p = 0.76	r = 0.14<br>p = 0.66			
Hbb-b1	r = -0.58<br>p = **0.004**	r = -0.13<br>p = 0.70	r = 0.14<br>p = 0.66	r = 0.50<br>p = **0.02**	r = 0.04<br>p = 0.92	r = 0.33<br>p = 0.30	r = 0.11<br>p = 0.60	r = -0.19<br>p = 0.57	r = -0.08<br>p =0.81
1500015O10Rik	r = -0.19<br>p = 0.38	r = 0.28<br>p = 0.41	r = 0.30<br>p = 0.35	r = 0.13<br>p = 0.55	r = -0.43<br>p = 0.19	r = 0.08<br>p = 0.81	r = -0.07<br>p = 0.76	r = -0.49<br>p = 0.13	r = -0.14<br>p = 0.66
Mgp	r = -0.43<br>p = **0.04**	r = 0.41<br>p = 0.21	r = -0.31<br>p = 0.33	r = 0.19<br>p = 0.38	r = -0.55<br>p = 0.08	r = 0.25<br>p = 0.43	r = 0.32<br>p = 0.14	r = -0.50<br>p = 0.12	r = 0.68<br>p = **0.01**

	Hbb-b1			*1500015O10Rik*		
	All animals	Control group	Stress group	All animals	Control group	Stress group
Hbb-b1	---			---		
1500015O10Rik	r = 0.59<br>p = **0.003**	r = 0.16<br>p = 0.63	r = 0.36<br>p = 0.26			
Mgp	r = 0.67<br>p = **0.0005**	r = 0.01<br>p = 0.96	r = 0.38<br>p = 0.23	r = 0.70<br>p = **0.000**	r = 0.89<br>p = **0.000**	r = 0.36<br>p = 0.25
